# Characterisation of graphene electrodes for microsystems and microfluidic devices

**DOI:** 10.1038/s41598-019-42108-x

**Published:** 2019-04-08

**Authors:** Michelle Del Rosso, C. Harrison Brodie, Saipriya Ramalingam, David M. Cabral, Erica Pensini, Ashutosh Singh, Christopher M. Collier

**Affiliations:** Applied Optics and Microsystems Laboratory, University of Guelph, Guelph, ON N1G 2W1 Canada

## Abstract

Fabrication of microsystems is traditionally achieved with photolithography. However, this fabrication technique can be expensive and non-ideal for integration with microfluidic systems. As such, graphene fabrication is explored as an alternative. This graphene fabrication can be achieved with graphite oxide undergoing optical exposure, using optical disc drives, to impose specified patterns and convert to graphene. This work characterises such a graphene fabrication, and provides fabrication, electrical, microfluidic, and scanning electron microscopy (SEM) characterisations. In the fabrication characterisation, a comparison is performed between traditional photolithography fabrication and the new graphene fabrication. (Graphene fabrication details are also provided.) Here, the minimum achievable feature size is identified and graphene fabrication is found to compare favourably with traditional photolithography fabrication. In the electrical characterisation, the resistivity of graphene is measured as a function of fabrication dose in the optical disc drive and saturation effects are noted. In the microfluidic characterisation, the wetting properties of graphene are shown through an investigation of the contact angle of a microdroplet positioned on a surface that is treated with varying fabrication dose. In the SEM characterisation, the observed effects in the previous characterisations are attributed to chemical or physical effects through measurement of SEM energy dispersive X-ray spectra and SEM images, respectively. Overall, graphene fabrication is revealed to be a viable option for development of microsystems and microfluidics.

## Introduction

Microsystems, or microelectromechanical systems (MEMS), make use of distinct and advantageous physical properties, including piezoelectric effects^1^, optoelectronic interaction^2^ thermal expansion^3^, and acoustic activation^4^. Strategic leveraging of these physical properties has led to microsystems becoming ubiquitous in today’s modern world. Further to this point, microsystems have found applications in the following industries: automotive, e.g., seatbelt activation with accelerometers^5^ and airbag switches^6^; biotechnology^7^, e.g., polymer chain reaction^8^ and advances in DNA and protein separations^8^; and healthcare^9^, e.g., disposable blood pressure sensors^10^ and point-of-care diagnostic devices^11^.

Point-of-care diagnostic devices are of particular interest—given their life saving potential—and such microsystems make use of microfluidic technologies. These microfluidic technologies enable microlitre and nanolitre fluid volumes to be actuated through microchannels, in the case of continuous-flow microfluidics, or between planar electrodes, in the case of digital (i.e., droplet-based) microfluidics^12^ and electrowetting-on-a-dielectric devices^13^.

Microsystems, and its subset of microfluidic technologies, share a desire for low cost and rapid fabrication of constituent components, as traditional photolithography fabrication can have debilitating associated cost. Specifically, there is great interest in developing low cost fabrication processes for microsystems and this is the subject of much recent research^14^. Here, it is desirable to achieve fabrication of highly-conductive electrodes with micro-scale feature sizes^15^ and to utilise materials with favourable wetting properties (i.e., possessing the ability to produce high contact angles for beaded microdroplets)^16^. The need for low-cost materials that lend themselves to use in microfluidic devices has led researchers to explore the novel material graphene^15,17–19^. Graphene can be fabricated in specified patterns, with graphite oxide undergoing optical exposure and conversion to graphene with simple optical disc drives, as reported previously^15^. Preliminary studies have shown favourable wetting properties^17^. However, there is still a gap in the literature for deep explorations into the fabrication of graphene (through comparison to traditional photolithography) and characterisation of graphene for use in microsystem and microfluidic electrodes (through electrical and microfluidic contact angle studies).

In this work, the above gap in the literature is addressed. An exhaustive characterisation of graphene fabrication is performed—with consideration to application in microfluidic electrodes. First, a fabrication characterisation is presented. Here, a comparison is performed between traditional photolithography fabrication and the new graphene fabrication. (Graphene fabrication details are also provided via the Appendix of this manuscript.) Here, the minimum achievable feature size is identified and graphene fabrication is found to compare favourably with traditional photolithography fabrication. Second, an electrical characterisation is presented. The resistivity of graphene is measured as a function of fabrication dose in the optical disc drive. The minimum fabrication dose required for saturation of resistivity is identified. Third, a microfluidic characterisation is presented. Here, the wetting properties of graphene are shown through an investigation of the contact angle of a microdroplet positioned on a surface that is treated with varying fabrication dose. Fourth, a scanning electron microscopy (SEM) characterisation is presented. The observed effects in the previous characterisations are attributed to chemical or physical effects through measurement and analysis of SEM energy dispersive X-ray spectra and SEM images, respectively. Ultimately, graphene fabrication is revealed to be a viable option for development of microsystems and microfluidics.

## Results

### Fabrication characterisation

**Figure 1:**
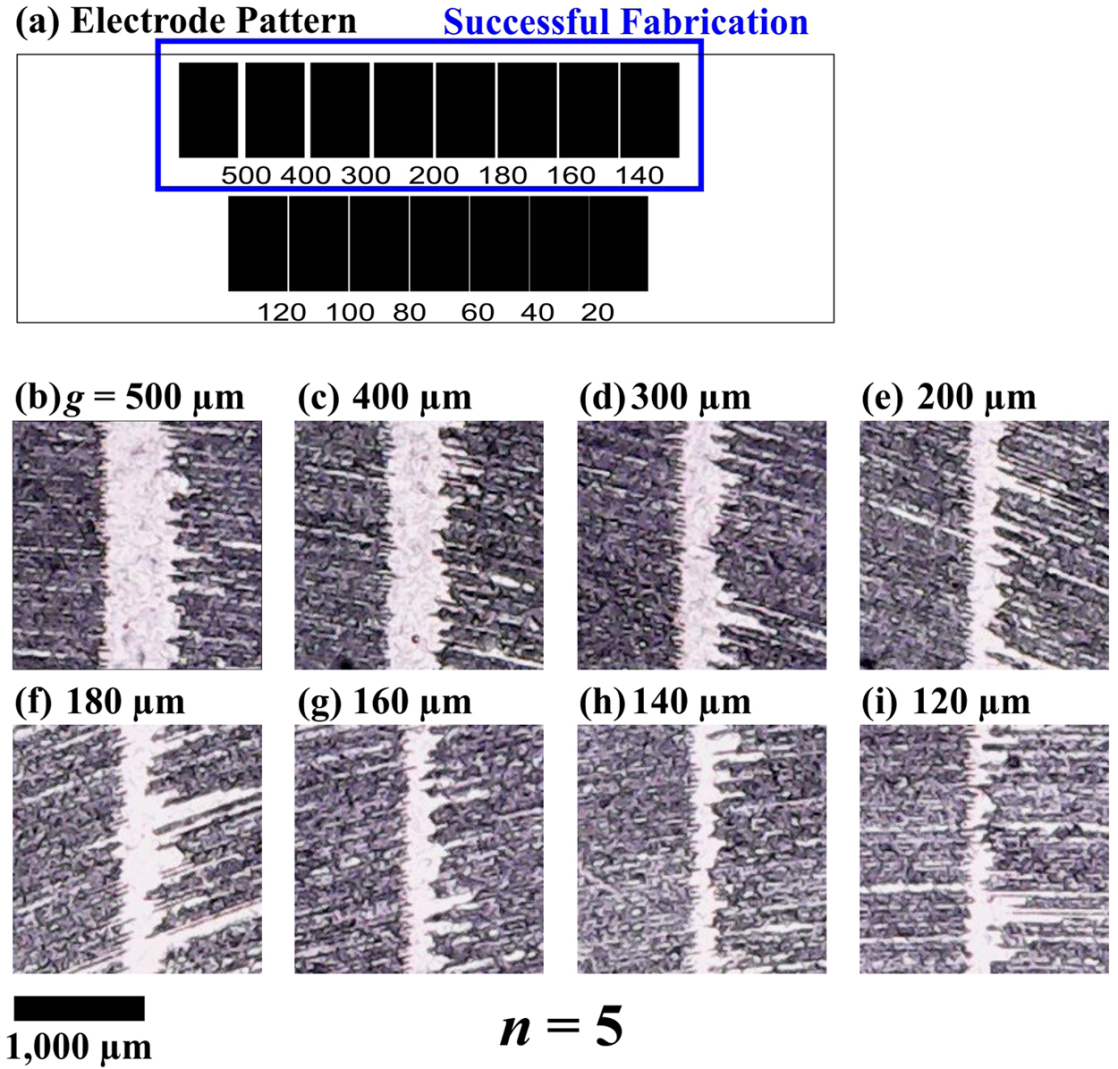
Graphene fabrication is shown with (**a**) the mask pattern developed to fabricate the graphene electrodes along with the images captured by the complementary metal-oxide-semiconductor (CMOS) image sensor of the (**b–i**) eight electrode gaps with decreasing sizes of *g* = 500, 400, 300, 200, 180, 160, 140, and 120 μm, respectively, at the optimal number of doses through the optical disc drive (*n* = 5).

To evaluate the (low cost and rapid) graphene fabrication method, minimum achievable feature size is considered. Once established, this minimum achievable feature size can be compared to that of traditionally photolithography fabrication, commonly used in fabrication of microsystems^20^. To establish this minimum achievable feature size, a recipe for graphene fabrication is followed, as described in the Appendix of this manuscript. For this fabrication characterisation, a mask pattern is developed (Fig. 1(a)) with decreasing electrodes gap sizes of *g* = 500, 400, 300, 200, 180, 160, 140, 120, 100, 80, 60, 40, and 20 μm. For the first eight of these electrode gap sizes, fabrication of the mask pattern is presented in Fig. 1(b–i), respectively. Here, there is successful feature separation down to 140 μm (Fig. 1(h)), with 120 μm (Fig. 1(i)) being unsuccessful, thereby establishing 140 μm as the minimum achievable feature size for the graphene fabrication. (The other smaller electrode gap sizes, *g* = 100 to 20 μm, are also unsuccessful and are omitted from Fig. 1).

**Figure 2:**
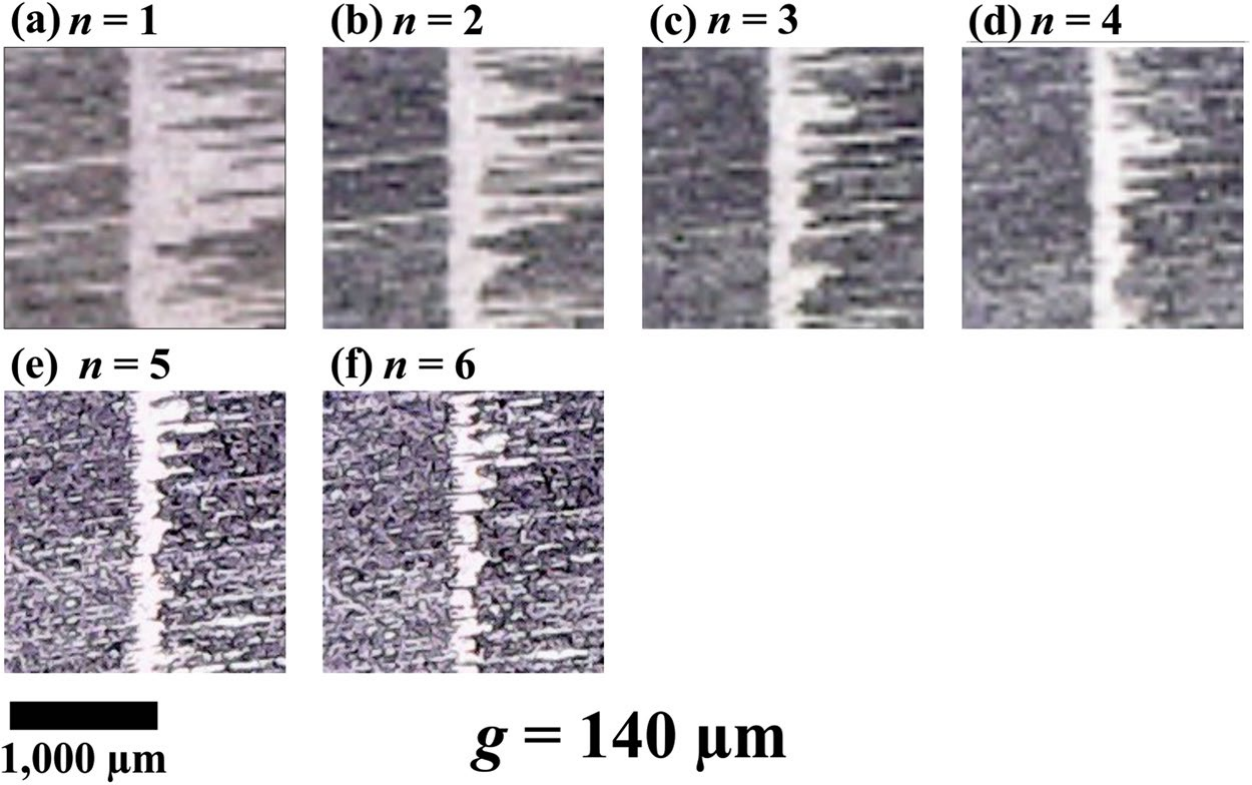
Six images of the 140 µm graphene electrode gap size captured by the CMOS image sensor at each cycle displaying the number of doses, *n* = 1–6, for (**a–f**) respectively. A dose of *n* = 5 is the optimal number of doses.

It should be noted that the implementation of the recipe for graphene fabrication revealed five doses, *n* = 5, through the optical disc drive to be ideal for distinguishing electrodes gaps. This conclusion is supported by Fig. 2 that shows graphene fabrication of 120 μm electrode gaps as a function of dose. It is clear that one to five doses yield increasingly clear electrodes, while six doses yield an over exposure with graphene appearing midgap (i.e., the conversion to graphene is pushed beyond the optimal dose and the intermediate graphite oxide becomes cross-contaminated with graphene).

**Figure 3:**
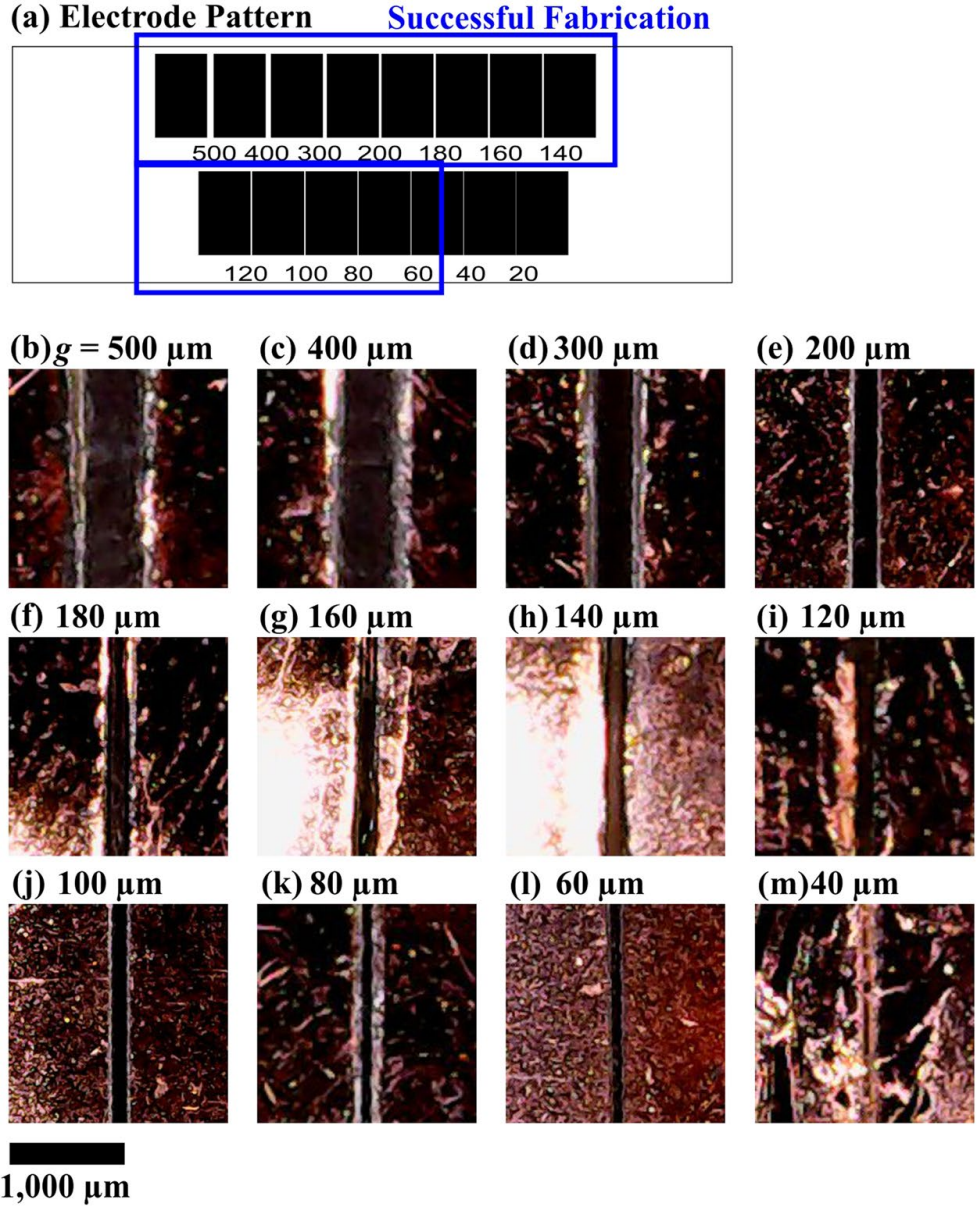
Traditional photolithography fabrication is shown with (**a**) the mask pattern developed to fabricate the photolithography electrodes along with the images captured by the CMOS image sensor of the (**b–m**) twelve electrode gaps with decreasing sizes of *g* = 500, 400, 300, 200, 180, 160, 140, 120, 100, 80, 60, and 40 μm, respectively.

For a benchmark comparison, the graphene fabrication is juxtaposed with traditional photolithography fabrication. The results of the traditional photolithography fabrication are presented in Fig. 3—with Fig. 3(a) showing the negative photolithography of the mask pattern from Fig. 1—with images shown for electrode gap sizes of *g* = 500 to 40 μm in Fig. 3(b–m), respectively. Here, there is successful feature separation down to *g* = 60 μm (Fig. 3(l)), with *g* = 40 μm (Fig. 3(m)) being unsuccessful, thereby establishing *g = *60 μm as the minimum achievable feature size for the traditional photolithography fabrication.

From the above results, it is clear that graphene fabrication, although achieving a marginally coarser minimum feature size (80 μm difference) than traditional photolithography fabrication, is a contender for fabrication of electrodes in microsystems (or in microfluidic devices). This is because graphene fabrication achieves micro-scale minimum feature sizes. It is shown in the next subsections that graphene fabrication can yield great utility for use in microfluidic devices.

### Electrical characterisation

**Figure 4:**
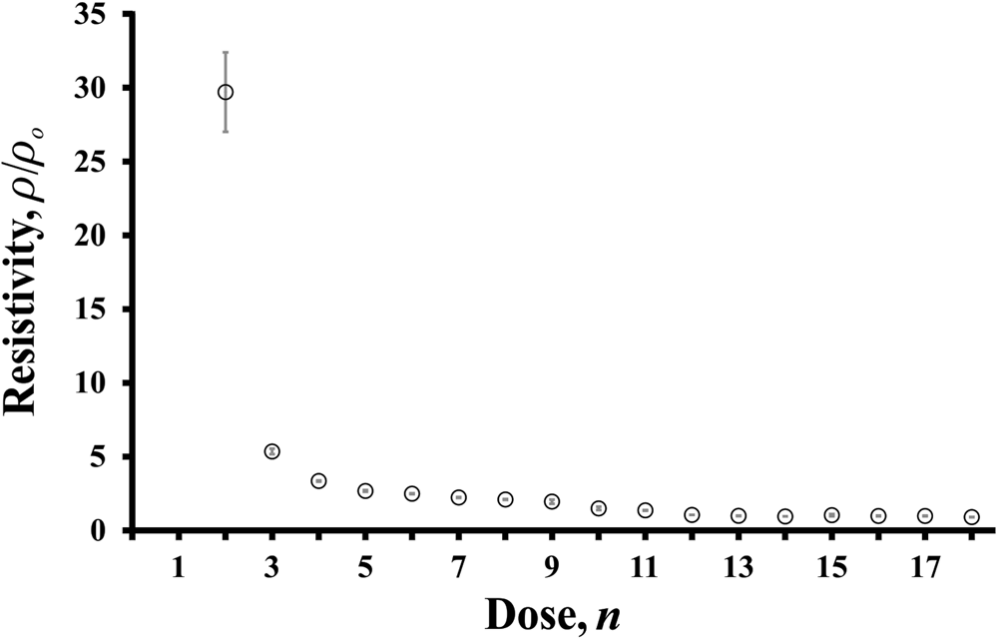
The (normalized) resistivity, *ρ*/*ρ*_0_, of the graphene electrodes as a function of the number of doses, *n*, through the optical disc drive.

Characterising the electrical properties of microsystems fabricated using the graphene fabrication method is of the utmost importance for use as electrodes in microsystems. Figure 4 displays the (normalized) results of the electrical characterisation with resistivity of the fabricated graphene, *ρ*, versus the number of doses through the optical disc drive, *n*. The error bars represent the standard deviation over three trials. The resistivity initially forms a high value (beyond the limit of our measurement) for *n* = 1, which indicates the insulative nature of the graphite oxide. The resistivity falls considerably for additional doses and reaches a steady-state resistivity, *ρ*_0_, after *n* = 12 doses, representing the final conversion of graphite oxide to graphene.

Given the ability to achieve (and even tune) the resistivity, graphene fabrication of electrodes is again shown to be a viable option for microsystems.

### Microfluidic characterisation

**Figure 5:**
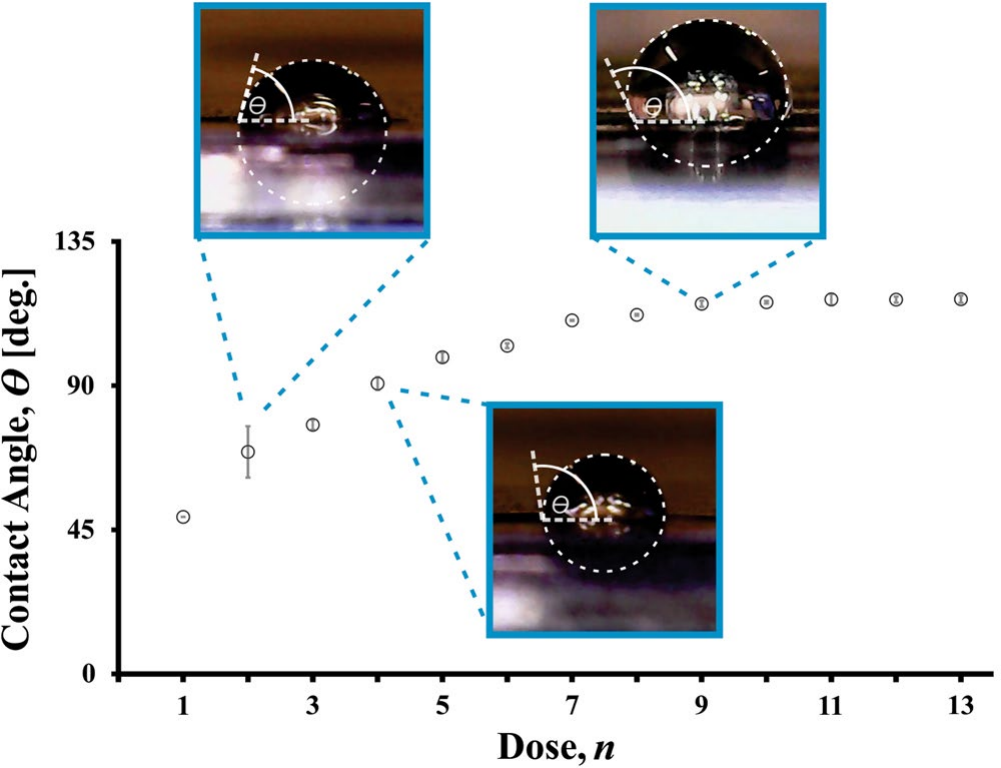
The contact angle, *θ*, of a water droplet on the graphene electrode as a function of the number of doses, *n*, through the optical disc drive. The inset images are captured using the CMOS image sensor, and are displayed for *n* = 2, 4, and 9, for low, medium, and high contact angle, respectively.

When fabricating electrodes for use in microsystems, particularly in microfluidic devices, it is desirable to select materials with favourable wetting properties such as a high contact angle (i.e., greater than 90°)^16^. To meet this desire, a detailed investigation of the wetting properties of the graphene fabrication method is performed through a microfluidic characterisation. The results of the microfluidic characterisation is presented in Fig. 5 as a graph of the contact angle, *θ*, versus the number of doses through the optical disc drive. For the microdroplets placed on the graphene surface, images of representative low, medium, and high contact angles are shown as insets for dose of *n* = 2, 4, and 9, respectively. (As shown in these insets, the contact angles are measured from the graphene surface to the outer most region of the microdroplet.) The contact angle increases monotonically from *θ* = 50° at a dose of *n* = 1 until it saturates to a contact angle of *θ* = 116° in the range of *n* = 7–9. This result is of particular importance for optofluidic applications^16^ within microfluidic devices, as graphene fabrication provides tunability of the contact angle over the contact angle range of 50° to 116°, without the application of any further hydrophobic materials (e.g., Teflon^12^), which is favourable compared to traditional photolithography fabrication.

### Scanning electron microscopy characterisation

An important clarification for the above characterisations is to distinguish what observations can be attributed to chemical changes (i.e., graphite oxide transitioning to graphene) and what observations can be attributed to physical changes (i.e., increased surface roughness). This important clarification can be addressed through a scanning electron microscopy (SEM) characterisation, whereby chemical changes are observed through SEM energy dispersive X-ray (EDX) spectra and physical changes are observed through SEM images.

**Figure 6:**
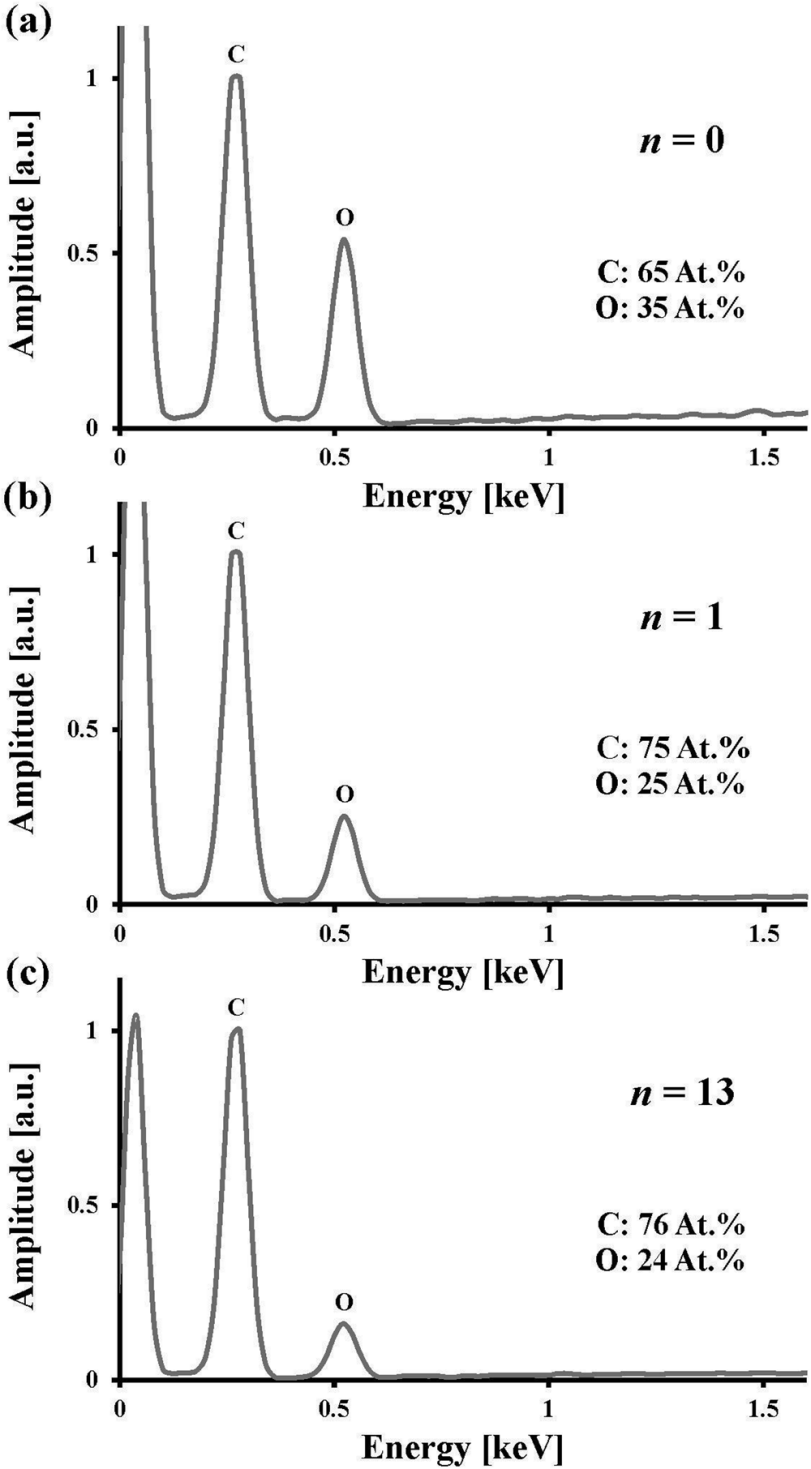
Scanning electron microscopy EDX spectra are shown for graphene for dose of *n* = 0, *n* = 1, and *n* = 13, representing graphite oxide prior to optical exposure, graphene after initial optical exposure, and graphene after extensive optical exposure, respectively. These are representative SEM EDX spectra. An exhaustive set of SEM EDX spectra measurements are taken to show atomic percent of carbon and oxygen of 65% and 35% for *n* = 0 and 76% and 24% for *n* = 1–13.

To attribute graphene effects to chemical changes, SEM EDX spectra are presented in Fig. 6. (The graphene fabrication process is followed to produce samples with dose of *n* = 0 through to *n* = 13, and each sample undergoes spectroscopy to produce SEM EDX spectra.) Representative SEM EDX spectra are shown for *n* = 0, 1, and 13 as these samples correspond to, respectively, graphite oxide prior to optical exposure, graphene after initial optical exposure, and graphene after extensive optical exposure. There is a transition from *n* = 0 (prior to optical exposure) to *n* = 1 (after initial optical exposure) that results in large change in atomic percent of carbon and oxygen of respective atomic percentages of 65% and 35% (the mean of four points on the sample for *n* = 0) to atomic percent of carbon and oxygen of respective atomic percentages of 76% and 24% which is maintained through to *n* = 13 (after extensive optical exposure) within an atomic percent standard deviation of 2%. (This atomic percent standard deviation is based on four trials at each of *n* = 1 through to *n* = 13). The overall trend follows previous observations of an increase and decrease in atomic percent of carbon and oxygen, respectively^21^. The large chemical change due to initial optical exposure mimics the large change in resistivity observed in Fig. 4 for the electrical characterisation. As such, this resistivity change from the Electrical Characterisation subsection can be attributed to the chemical change of the transition from graphite oxide to graphene.

**Figure 7:**
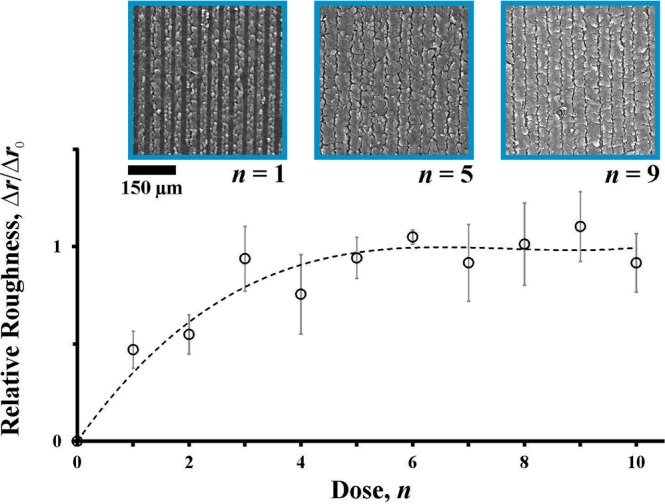
An SEM image analysis is shown with normalized relative roughness, Δ*r*/Δ*r*_0_, versus dose, *n*. The normalized relative roughness begins to saturate in the range of *n* = 7–9. The saturation value of the relative roughness, Δ*r*_0_, is estimated to be 4.4. The curve of best fit is shown in a dashed line with agreement of *R*^2^ = 0.91 with the experimental data.

To attribute graphene effects to physical changes, an SEM image analysis is presented in Fig. 7. Here, SEM images are converted to topographical surface plots. From these topographical surface plots, surface areas are extracted. This analysis follows a previously reported method^22^. The surface roughness, *r*, then can be found as the surface area divided by the top view area of interest. This surface roughness can be divided into two parts through *r* = 1 + Δ*r*, where the unit value is the surface roughness of a smooth surface and the relative roughness, Δ*r*, quantifies the increased roughness beyond the smooth surface. In Fig. 7, the normalized relative roughness, Δ*r*/Δ*r*_0_, is shown versus dose, *n*. (The saturation value of the relative roughness is Δ*r*_0_.) It can be seen that there is an overall increasing trend for *n* = 0–6. The normalized relative roughness begins to stabilize in the range of *n* = 7–9 and this mimics the saturation of contact angle observed in Fig. 5 in the microfluidic characterisation. As such, these contact angle effects from the Microfluidic Characterisation subsection can be attributed to physical changes (i.e., increased surface roughness with increased optical exposure of the surface during optical disc drive lithography). This observation is consistent with previous reports correlating increased surface roughness with increased contact angle of microdroplets^23^.

## Conclusions

A graphene fabrication process was characterised for use in microsystems and microfluidic devices. The graphene fabrication process was based on graphite oxide undergoing optical exposure through an optical disc drive and converting to graphene. Fabrication, electrical, microfluidic, and scanning electron microscopy characterisations of the graphene fabrication yielded micro-scale feature sizes, low resistivity properties, tunable wetting properties, and provided insight into chemical and physical graphene effects, respectively. The resistivity of graphene showed a close connection to chemical composition and the contact angle of microdroplets on graphene showed a close connection to physical composition of the surface. Overall the graphene fabrication process compared favorably to a photolithography fabrication process.

## Methods

### Graphene fabrication procedure

The graphene fabrication procedure begins with a graphite oxide solution, made by mixing 100 mg of graphite oxide powder (ACS Materials) with 27 mL of deionized water in a beaker placed into a bath sonicator (Fisherbrand M series) for 15 minutes. To prepare for exposure, a film of transparent plastic material in the shape of an optical disc is adhered onto a LightScribe disc. A volume of 17 mL of the graphite oxide solution is applied to the LightScribe disc with a syringe and left to air dry for 48 hrs. The mask pattern (Fig. 3(a)) designed in image processing software is uploaded to the LightScribe software to be burned onto the LightScribe disc with a laser with wavelength of 780 nm. The LightScribe disc that is coated in graphite oxide is then placed into the LightScribe drive, and the pattern is optically exposed into the graphite oxide to form graphene in the exposed locations. To increase the dose number, the optical exposure of the LightScribe disc is repeated for the desired number of doses.

### Traditional photolithography fabrication procedure

For the traditional photolithography fabrication, the desired mask pattern is made using image processing software with feature sizes as small as 5 µm. To make a negative photomask, the image file is exported from the image processing software as a portable document format file with a rendering resolution of 10,000 dots per inch and printed with black emulsion onto a film of transparent plastic material.

To prepare the substrate, a 75 mm × 25 mm × 1 mm glass slide with a 50 nm copper coating is cleaned of any debris. The copper-coated glass slide then undergoes a spincoating process to coat with negative photoresist. The copper-coated glass slide is placed into a programmable spincoater (CEE 200X), and approximately 3 mL of negative photoresist (MICROCHEM SU-8) is dispensed manually onto the surface using a syringe. The programmable spin coater is set to leave a 30 µm layer of the negative photoresist through a process of 500 rpm for 25 seconds with acceleration of 100 rpm/s and 1300 rpm for 30 seconds at an acceleration of 300 rpm/s. The copper-coated glass slide then undergoes a soft bake process. The copper-coated glass slide is placed onto a hotplate (Corning PC351) for 90 seconds, and then immediately into an oven (Fisher Isotemp Vacuum Oven 282) for 5.5 min and then left to cool for 15 min. Once the copper-coated glass slide is cool, the negative photomask is placed ink-side-down onto the surface of the slide coated with SU-8 and loaded into an ultraviolet exposure chamber (KLOÉ UV KUB2) for an exposure of 100% energy for 7 seconds. The length of exposure time is found through

1 $$Duration=\frac{1}{23}Exposure\,Energy(\frac{mJ}{c{m}^{2}})$$

where duration is the exposure time in seconds and exposure energy is determined from the SU-8 negative photoresist data sheet. The copper-coated glass slide then undergoes a second soft bake process (identical to the first soft bake process). Once the copper-coated glass slide is cool, it is submerged in (agitated) SU-8 developer solution (MICROCHEM) for 1 minute, cleaned with a wipe, and left to air dry. With the copper exposed in the desired areas for removal, the slide is placed into a Ferric Chloride (MG Chemicals 415) solution for 3 seconds, and into a deionized water bath for 3 seconds. These last steps are repeated until the exposed areas are fully removed of copper.
